# 中性粒细胞/淋巴细胞比值预测根治性切除肺腺癌患者预后分析

**DOI:** 10.3779/j.issn.1009-3419.2018.08.04

**Published:** 2018-08-20

**Authors:** 高祥 王, 燃 熊, 汉然 吴, 广文 徐, 彩伟 李, 效辉 孙, 明然 解

**Affiliations:** 230001 合肥，安徽医科大学附属省立医院胸外科 Department of Thoracic Surgery, Anhui Provincial Hospital Affiliated to Anhui Medical University, Hefei 230001, China

**Keywords:** 肺肿瘤, 中性粒细胞与淋巴细胞比值, 生存, 预后, Lung neoplasms, Neutrophil-to-lymphocyte ratio, Survival, Prognosis

## Abstract

**背景与目的:**

已有的研究表明中性粒细胞计数/淋巴细胞计数比（neutrophil-to-lymphocyte ratio, NLR）对胃癌、结直肠癌、胰腺癌等多种恶性肿瘤的预后有显著影响，但是其对可切除肺腺癌患者预后影响目前研究较少。本研究旨在分析NLR与肺腺癌患者术后临床病理特征及预后的关系。

**方法:**

回顾性分析163例经病理确诊为肺腺癌并行肺腺癌根治切除术患者的临床随访病理资料。根据术前1周内化验结果计算NLR值。通过对受试者工作特征曲线（receiver operating characteristic curve, ROC）的分析得出NLR的临界值（cut-off值）。采用*Kaplan-Meier*生存曲线和*Cox*比例风险模型研究NLR对肺腺癌术后患者预后的影响。

**结果:**

采用5年生存作为终点绘制NLR值的ROC曲线，当NLR值为2.96时，正确指数（*Youden*指数）最大，敏感度为77.5%，特异度为75.9%。低NLR组累计5年生存率显著高于高NLR组（*P* < 0.05）。单因素及多因素生存分析显示：TNM分期和NLR水平与生存率显著相关（*P* < 0.05）。

**结论:**

NLR值作为一项便捷且有效的指标，可用于初步判断肺腺癌术后患者预后状况。

## Abstract (en)

**Background and objective:**

Previous studies have shown that the neutrophil-to-lymphocyte ratio (NLR) has a significant impact on the prognosis of many malignant tumors such as gastric cancer, colorectal cancer and pancreatic cancer, but the study on the prognosis of patients with resectable lung adenocarcinoma is less. The aim of this study is to investigate the correlation between the NLR and the clinicopathologic features of adenocarcinoma of lung patients who underwent radical pneumonectomy. Furthermore, this study aimed to clarify the predictive and prognostic significance of NLR in patients who underwent pneumonectomy for lung adenocarcinoma.

**Methods:**

This study reviewed the medical records of 163 patients with lung adenocarcinoma who underwent pneumonectomy. The receiver operating characteristic (ROC) curve and *Youden* index were used to determine the cut-off value of the NLR. Survival curves were described by *Kaplan-Meier* method and compared by *Log-rank* test. The univariate and multivariate analyses were performed with the *Cox* proportional hazard model to identify the prognostic factors.

**Results:**

When the NLR value was 2.96, the *Youden* index was maximal, with a sensitivity of 77.5% and a specificity of 75.9%. The 5-year survival rate in the low NLR group was higher than that in the high NLR group (*P* < 0.05). The univariate and multivariate analyses showed that TNM staging and NLR were independent factors in predicting survival rate.

**Conclusion:**

The NLR value was a simple and useful tool to predict the prognosis of lung adenocarcinoma after radical pneumonectomy.

肺癌发病率和死亡率在全世界范围内一直居于所有恶性肿瘤之首^[1, 2]^。肺癌的组织学类型包括小细胞肺癌和非小细胞肺癌，其中非小细胞肺癌占80%以上，腺癌是非小细胞肺癌中最常见的病理类型^[3, 4]^。腺癌早期即可侵犯血管、淋巴管，常在原发瘤引起症状前即已转移。手术是可切除非小细胞肺癌的主要治疗手段，但单一手术治疗对大多数肺癌患者是不够的。因此，如何挑选出术后复发和转移的高危患者并给予适当的临床干预是学术界研究热点之一。肿瘤-淋巴结-转移（tumor-node-metastasis, TNM）分期是临床上最常用的肺癌预后评价指标，但相同TNM分期患者的预后却不尽相同。近年来，越来越多的生物学指标和数学模型被用于肺癌患者预后的预测并指导临床的治疗^[5, 6]^。但很多生物学指标检测价格昂贵，不能作为常规检查而广泛使用。

外周血中性粒细胞计数与外周血淋巴细胞计数比（neutrophil-to-lymphocyte ratio, NLR）最早于1995年由Satoml提出，其计算公式为NLR=外周血中性粒细胞计数（×10^9^/L）/外周血淋巴细胞计数（×10^9^/L）。文献^[7-11]^报道，NLR对胃癌、结直肠癌、胰腺癌等多种恶性肿瘤的预后有显著影响，更高的NLR值提示预后不良。但其对可切除肺腺癌患者预后是否有影响，目前研究较少。本研究收集安徽医科大学附属省立医院2011年1月-2012年12月术后病理诊断为肺腺癌患者共163例，分析其NLR值并进行分组，比较分析两组患者临床病理资料、中位生存期和远期生存率情况。

## 1 资料与方法

### 1.1 一般资料

本研究选取安徽医科大学附属省立医院胸外科2011年1月-2012年12月接受肺癌根治术，术后病理证实为肺腺癌患者342例。纳入标准：①术后病理证实为肺腺癌；②接受系统性纵隔淋巴结清扫；③手术为肺叶切除、联合肺叶切除或全肺切除，并为R0切除（肿瘤完全切除，没有残留）。排除标准：①R1（病理学显微镜下看到有切缘肿瘤的残留）或R2（肉眼就可以看到肿瘤的残留）切除患者；②术前接受过新辅助治疗；③接受亚肺叶切除或未行系统性淋巴结清扫；④病例资料不完整。

基于以上标准，共163例患者纳入本研究。其中男性94例，女性69例；Ⅰ期64例，Ⅱ期37例，Ⅲ期62例。

治疗前检查包括：胸、上腹部计算机断层扫描（computed tomography, CT）增强、颅脑磁共振检查（magnetic resonance, MR）（平扫+增强）、骨扫描、电子支气管镜、心电图、肺功能，年龄 > 65岁患者加做超声心动图，术前化验为常规。肿瘤分期采用国际肺癌研究协会（International Association for the Study of Lung Cancer, IASLC）第8版TNM分期系统。

### 1.2 NLR计算方法

所有患者均在手术前1周内抽血检测血常规和生化检查，根据其结果，计算NLR值，计算公式为NLR=外周血中性粒细胞计数（×10^9^/L）/外周血淋巴细胞计数（×10^9^/L）。

### 1.3 治疗方法

手术方式包括肺叶切除150例，全肺切除13例。肺叶切除病例中包括单纯肺叶切除112例，袖式切除14例，联合肺叶切除24例。术中根据淋巴结分布图，系统清扫N1和N2站淋巴结。右肺癌清扫第2R、4R、7、8、9组纵隔淋巴结及肺叶间淋巴结，左肺癌清扫4组-9组纵隔淋巴结及肺叶间淋巴结。术后56例（34.4%）接受辅助性化疗，其中，48例接受4个周期足量化疗，8例因不能耐受或主观拒绝未能完成4个周期化疗。主要化疗方案包括：多西他赛+铂类、培美曲塞+铂类、长春瑞滨+铂类、吉西他滨+铂类等。7例（4.3%）患者术后接受辅助性放疗。

### 1.4 观察指标

观察术前NLR水平与患者临床病理资料的关系，和术前NLR水平对肺腺癌患者5年生存率的影响。

### 1.5 随访及统计学方法

采用门诊定期复诊和电话随访两种方式进行随访，术后第1年每3个月随访1次，第2年每半年随访1次，从第3年起每年随访1次，获取相关临床信息（包括胸、脑CT，骨扫描，腹部和肾上腺超声等）和患者生存情况。应用SPSS 24.0统计学软件进行数据分析，应用卡方检验比较两组患者临床病理资料。通过以5年生存为终点绘制受试者工作特征曲线（receiver operating characteristic curve, ROC）来评估5年总生存时间的敏感性和特异性，并通过计算正确指数（*Youden*指数）来决定NLR的最佳临界值（cut-off值）（图 1），然后以最佳cut-off值分为高NLR组和低NLR组。生存率根据*Kaplan-Meier*法计算，以*Log-rank*检验分析组间生存率的差异并作趋势检验。采用*Cox*模型进行多因素生存分析。*P* < 0.05为差异有统计学意义。

**1:**
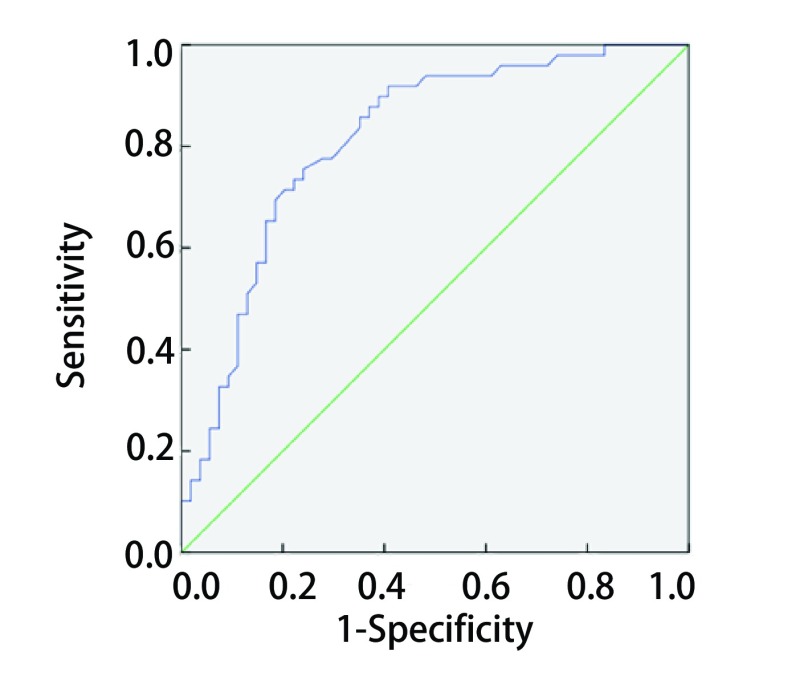
ROC曲线选取最佳NLR值 Receiver operating curve (ROC) analysis for the optimal cut-off value of neutrophil-to-lymphocyte ratio (NLR)

## 2 结果

### 2.1 NLR临界值确定

通过5年生存率作为终点来绘制NLR的ROC（图 1），并通过计算*Youden*指数来确定最佳cut-off值，*Youden*指数=敏感度-（1-特异度）。ROC下面积为0.814，当NLR值为2.96时，敏感度为77.5%，特异度为75.9%，*Youden*指数最大为0.534。因此，本研究将2.96作为最佳cut-off值，NLR≥2.96作为高NLR组，NLR < 2.96作为低NLR组。

### 2.2 NLR与患者临床病理特征关系

全组患者NLR的平均值为3.30，最小为1.80，最大为6.87。两组患者性别、年龄、吸烟史、外周血中性粒细胞计数、外周血淋巴细胞计数和有无接受放疗方面无明显差异（*P* > 0.05）。相对于低NLR组患者，高NLR组患者的TNM分期更晚，肿瘤直径更长、接受辅助性化疗的更多（*P* < 0.05）（表 1）。

**1:** NLR与患者临床病理特征关系 Relationship between NLR and the clinicopathological features of the patients

Characteristics	High NLR group (*n*=57)	Low NLR group (*n*=106)	*χ*^2^	*P*
Gender			2.907	0.088
Male	38	56		
Female	19	50		
Age (yr)			2.182	0.140
≤65	38	82		
> 65	19	24		
Tumor diameter (cm)			4.071	0.044
≤3	35	81		
> 3	22	25		
TNM stage			9.734	0.008
Ⅰ	14	50		
Ⅱ	13	24		
Ⅲ	30	32		
Smoking			3.046	0.081
Yes	25	32		
No	32	74		
Operative approach			0.110	0.741
Lobectomy	53	97		
Pneumonectomy	4	9		
Chemotherapy			1.397	0.237
Yes	23	33		
No	34	73		
Radiotherapy			0.727	0.393
Yes	3	4		
No	103	53		
Neutrophil count (×10^9^)			0.672	0.412
≥6.5	6	6		
< 6.5	51	100		
Lymphocyte count (×10^9^)			0.240	0.624
≥1	49	88		
< 1	8	18		
TNM: tumor-node-metastasis; NLR: neutrophil-to-lymphocyte ratio.				

### 2.3 NLR和患者5年生存率的关系（图 2）

**2:**
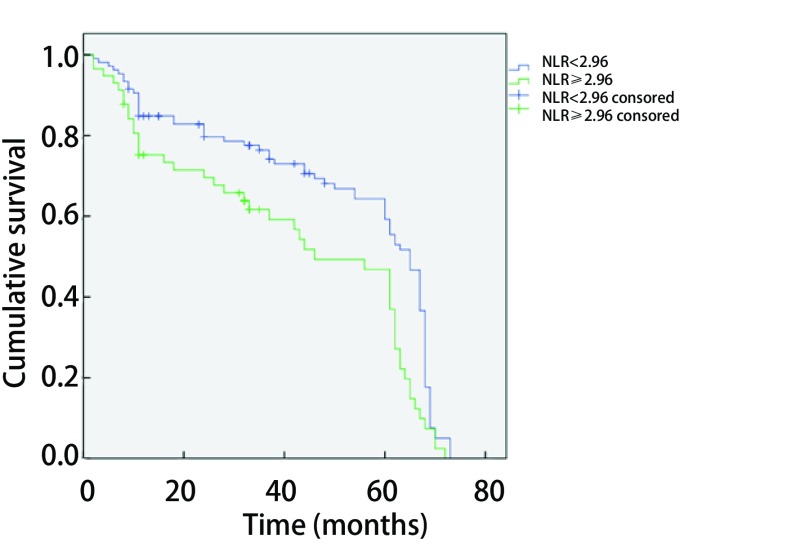
高组NLR与低组NLR生存曲线比较 *Kaplan-Meier* curves of survival rate according to NLR

 全组163例患者，随访过程中32例患者失访，但163例患者都纳入单、多因素分析。随访时间从2011年1月-2017年10月，总随访时间为82.0个月，中位随访时间68.0个月。全组患者中位生存期和第1年、3年、5年生存率分别为62.0个月和81.6%、72.4%、60.7%。高NLR组中位生存期和第1年、3年、5年生存率分别为52.0个月和73.3%、59.2%、37.0%。低NLR组中位生存期和第1年、3年、5年生存率分别为65.0个月和82.8%、74.1%、59.3%。低NLR组生存率明显优于高NLR组，差异有统计学意义（*P*=0.001）。

### 2.4 影响患者预后的单因素及多因素分析

将患者临床病理资料分别进行单因素分析发现，肿瘤最大径、TNM分期、辅助性化疗和NLR水平与患者生存率显著相关（*P* < 0.05）（表 2）。将患者临床病理资料代入*Cox*模型进行多因素分析，结果显示TNM分期和NLR水平是影响患者生存率的独立预后因素（*P* < 0.05）（表 3）。

**2:** 影响生存率的预后因素的单因素分析 Univariate analysis of prognostic factors influencing survival rate

Characteristics	Number	Median survival time (month)(95%CI)	5-yr survival rate (%)	*P*
Gender				0.277
Male	94	61.0 (58.5-63.5)	57.3	
Female	69	61.0 (55.8-66.2)	52.3	
Age (yr)				0.430
≤65	120	61.0 (52.9-69.1)	53.4	
> 65	43	62.0 (58.4-65.6)	58.7	
Tumor diameter (cm)				0.003
≤3	116	62.0 (59.4-64.6)	61.3	
> 3	47	44.0 (27.6-60.4)	38.6	
TNM stage				< 0.001
Ⅰ	64	65.0 (62.1-67.9)	63.3	
Ⅱ	37	62.0 (54.2-69.8)	54.8	
Ⅲ	62	42.0 (29.0-55.0)	34.7	
Smoking				0.623
Yes	57	62.0 (60.1-63.9)	63.0	
No	106	61.0 (55.9-66.1)	59.4	
Operative approach				0.357
Lobectomy	150	62.0 (59.7-64.2)	58.9	
Pneumonectomy	13	50.0 (19.1-80.9)	53.2	
Chemotherapy				0.040
Yes	56	46.0 (21.2-70.8)	36.4	
No	107	62.0 (59.5-62.5)	57.5	
Radiotherapy				0.874
Yes	7	54.0 (27.7-80.3)	28.6	
No	156	61.0 (59.5-62.5)	55.7	
Neutrophil count (×10^9^)				0.318
≥6.5	12	37.0 (6.5-67.5)	25.0	
< 6.5	151	62.0 (59.4-62.6)	56.1	
Lymphocyte count (×10^9^)				0.226
≥1	137	62.0 (59.7-64.3)	60.0	
< 1	26	46.0 (33.8-58.2)	26.9	
NLR				0.001
≥2.96	57	46.0 (32.3-59.7)	37.0	
< 2.96	106	65.0 (61.6-68.4)	59.3	

**3:** 影响生存率的预后因素的多因素分析 Multivariate analysis of prognostic factors influencing survival rate

Characteristics	*P*	HR (95%CI)
NLR	0.025	1.540 (1.055-2.247)
TNM stage	< 0.001	1.550 (1.250-1.921)
Gender	0.780	1.223 (0.813-1.838)
Age	0.644	0.860 (0.568-1.303)
Smoking	0.834	0.937 (0.667-1.453)
Tumor diameter	0.205	1.395 (0.905-2.149)
Operative approach	0.719	0.998 (0.455-2.188)
Chemotherapy	0.479	0.826 (0.545-1.254)
Radiotherapy	0.678	1.296 (0.600-2.801)
Neutrophil count	0.756	0.940 (0.537-1.644)
Lymphocyte count	0.937	0.734 (0.336-1.601)

## 3 讨论

如何判断肺腺癌患者的预后，筛选出复发和转移的高危患者，并对其实施早期干预，是临床医师面临的重大课题之一。近年来，研究^[12-14]^表明系统性炎症反应与多种实体肿瘤的预后相关。外周血白细胞亚群计数，尤其是中性粒细胞的增加和淋巴细胞的减少，可反映系统性炎症反应的状态。NLR是由外周血中性粒细胞总数和外周血淋巴细胞总数计算得出，其结果简单易得。文献^[7-11]^报道NLR对胃癌、结直肠癌、胰腺癌等多种恶性肿瘤的预后有显著影响。本研究发现，术前NLR水平与根治性切除的肺腺癌患者的远期生存率显著相关，术前NLR水平高的患者的远期生存率明显降低。尽管TNM分期是可切除肺腺癌的主要预后判断标准，但多因素预后分析结果显示，术前NLR水平也是能够很好判断患者预后的独立因素。

肿瘤进展引起的系统性炎症反应会影响患者的免疫状态。免疫状态低下加速了肿瘤的进展，而肿瘤进展引起的恶性消耗进一步影响患者的免疫状态，形成一个恶性循环。文献报道，在胃癌、结直肠癌和胰腺癌的研究中，术前高NLR是影响患者远期生存的独立预后因素。本研究发现，术前高NLR组患者的中位生存期和5年生存率明显差于低NLR组。Chen等^[15]^研究发现，新辅助化疗的晚期胃癌患者中低NLR组的1年、3年、5年生存率均高于高NLR组。Gao等^[16]^研究1, 281例食管鳞状细胞癌发现，NLR水平是食管鳞状细胞癌患者预后的独立因素，NLR水平升高预示着不良的预后。我们认为其原因可能在于：①中性粒细胞增加能够重建肿瘤细胞外基质的功能，从而促进肿瘤的生长和转移；②中性粒细胞能够抑制淋巴细胞对肿瘤细胞毒性作用和抑制T细胞扩散等功能；③淋巴细胞减少使其溶解和杀死肿瘤细胞的活性降低，进而促进肿瘤细胞的增值和迁移。因此肺腺癌患者中，NLR水平升高显示免疫学反应在促进肿瘤发展和抗肿瘤中的一种不平衡状态。

中性粒细胞是白细胞主要组成成分，除了在消灭肿瘤细胞中发挥作用外，在肿瘤生长刺激中也起着重要作用，其主要通过分泌不同的细胞因子、生长因子、蛋白酶及其他分子来发挥作用^[17]^。较高的中性粒细胞计数预示着体内炎症较重，向着促进肿瘤方向进展。因此，较高的中性粒细胞计数预示着不良的预后。淋巴细胞是肿瘤细胞免疫调节的重要因素，对肺腺癌患者来说，较低的外周血淋巴细胞计数预示着不良的预后。但这两个指标的敏感性均不强，本组病例中，大多数患者术前的总中性粒细胞计数和总淋巴细胞计数都是正常的。因此，相对于单纯的外周血中性粒细胞计数或外周血淋巴结细胞计数监测，术前NLR水平是评估肺腺癌患者预后更佳的临床指标。

关于NLR的cut-off值，有的学者借鉴其他文献中的cut-off值，亦有学者使用术前中位数来作为cut-off值。文献^[18-23]^报道，NLR的cut-off值一般在2-5之间，不同肿瘤或不同地域的人群均存在异质性，其结果均对最佳cut-off值会有影响。另外，研究对数据的采集、整理、计算等存在各种因素的影响，因此采用中位数方法得到最佳cut-off值也是不够严谨的。本研究通过绘制ROC曲线来评估5年生存率的敏感性及特异性，并通过计算*Youden*指数来决定NLR的最佳cut-off值，其研究结果更为可靠。

综上所述，NLR水平与根治性手术肺腺癌患者的预后显著相关，术前高NLR的患者术后的远期生存率低于术前低NLR的患者。因此，对术前高NLR的肺腺癌患者应予以更加密切的关注，及时干预肿瘤复发和转移，以求获得更好的预后。但是本研究是单中心回顾性分析，存在一定的病例选择性偏倚。另一方面，本研究的样本数较少，仍需要大样本前瞻性研究来证实。
